# Improved Clinical Outcomes During Long-term Osilodrostat Treatment of Cushing Disease With Normalization of Late-night Salivary Cortisol and Urinary Free Cortisol

**DOI:** 10.1210/jendso/bvae201

**Published:** 2024-11-12

**Authors:** John Newell-Price, Maria Fleseriu, Rosario Pivonello, Richard A Feelders, Mônica R Gadelha, André Lacroix, Przemysław Witek, Anthony P Heaney, Andrea Piacentini, Alberto M Pedroncelli, Beverly M K Biller

**Affiliations:** The School of Medicine and Population Health, University of Sheffield, Sheffield S10 2RX, UK; Pituitary Center, Departments of Medicine and Neurological Surgery, Oregon Health & Science University, Portland, OR 97239, USA; Dipartimento di Medicina Clinica e Chirurgia, Sezione di Endocrinologia, Università Federico II di Napoli, 80138 Naples, Italy; Department of Internal Medicine, Endocrine Section, Erasmus Medical Center, 3015 GD Rotterdam, Netherlands; Neuroendocrinology Research Center, Endocrinology Section, Medical School and Hospital Universitário Clementino Fraga Filho, Universidade Federal do Rio de Janeiro, Rio de Janeiro 21941-617, Brazil; Department of Medicine, Centre hospitalier de l’Université de Montréal, Montreal, QC H2X 0A9, Canada; Department of Internal Medicine, Endocrinology and Diabetes, Medical University of Warsaw, 02-091 Warsaw, Poland; Division of Endocrinology, Diabetes and Metabolism, Department of Medicine, David Geffen School of Medicine, University of California, Los Angeles, CA 90095, USA; Recordati SpA, 20148 Milan, Italy; Recordati AG, 4057 Basel, Switzerland; Neuroendocrine and Pituitary Tumor Clinical Center, Massachusetts General Hospital, Boston, MA 02114, USA

**Keywords:** Cushing disease, osilodrostat, cortisol normalization, hypercortisolism, long-term treatment

## Abstract

**Purpose:**

To assess whether simultaneous normalization of late-night salivary cortisol (LNSC) and mean urinary free cortisol (mUFC) in patients with Cushing disease treated with osilodrostat is associated with better clinical outcomes than control of mUFC or LNSC alone.

**Methods:**

Pooled data from two phase III osilodrostat studies (LINC 3 and LINC 4) were analyzed. Both comprised a 48-week core phase and an optional open-label extension. Changes in cardiovascular/metabolic-related parameters, physical manifestations of hypercortisolism, and quality of life (QoL) were evaluated across the following patient subgroups: both LNSC and mUFC controlled, only mUFC controlled, only LNSC controlled, and neither controlled.

**Results:**

Of 160 patients included in the analysis, 85.0% had both LNSC and mUFC uncontrolled at baseline. At week 72, 48.6% of patients had both LNSC and mUFC controlled; these patients generally exhibited greater improvements in cardiovascular/metabolic-related parameters than those with only mUFC controlled or both LNSC and mUFC uncontrolled: systolic/diastolic blood pressure, −7.4%/−4.9%, −6.0%/−5.5%, and 2.3%/0.8%, respectively; fasting plasma glucose, −5.0%, −4.8%, and 1.9%; glycated hemoglobin, −5.1%, −4.8%, and −1.3%. Weight, waist circumference, and body mass index improved with control of LNSC and/or mUFC; physical manifestations of hypercortisolism generally improved regardless of LNSC/mUFC control. Patients with both LNSC and mUFC controlled or only mUFC controlled had the greatest improvement from baseline to week 72 in QoL.

**Conclusion:**

In osilodrostat-treated patients with Cushing disease, normalization of LNSC and mUFC led to improvements in long-term outcomes, indicating that treatment should aim for normalization of both parameters for optimal patient outcomes.

**Clinical trial identifiers:**

NCT02180217 (LINC 3); NCT02697734 (LINC 4)

**Plain Language Summary**

**Why was this analysis carried out?**

- People with Cushing disease have higher-than-normal levels of the adrenal hormone cortisol, caused by excess production of ACTH by an adenoma (noncancerous tumor) on the pituitary gland.
- In people with Cushing disease, excess cortisol causes a range of symptoms, including physical changes such as stretch marks, bruising, a round face, and muscle wasting. They are also more likely to have cardiovascular conditions (such as high blood pressure), metabolic conditions (such as obesity and diabetes), and worse quality of life.
- Osilodrostat is a medicine used to treat people with Cushing disease. It reduces their cortisol levels and improves their symptoms and quality of life.
- In healthy individuals, cortisol levels change throughout the day with the circadian rhythm (internal body clock), with higher levels in the morning and lower levels late at night. In people with Cushing disease, this rhythm is lost, with high levels throughout the 24-­hour period or at bedtime and increased daily production.
- Doctors measure cortisol levels in different ways, for example, from a saliva sample taken late at night that assesses the circadian rhythm of cortisol levels or from a urine sample, which determines the total amount of cortisol produced over 24 hours. This analysis assessed whether patients in whom both late-night salivary cortisol and 24-hour urinary cortisol were controlled (reduced to normal levels) had better outcomes than those who had only 1 of these controlled.

**How was this analysis carried out?**

- The results of 2 osilodrostat studies were combined to allow a larger number of people to be included in this analysis.
- Clinical outcomes were compared in 4 groups of people: those in whom both late-night salivary and urinary cortisol were controlled; those who achieved control of urinary cortisol only; those who achieved control of salivary cortisol only; and those in whom neither were controlled.
- The outcomes of interest were cardiovascular/metabolic measures, physical changes, and quality of life.

**What were the overall results?**

- Before being treated with osilodrostat, most people had uncontrolled levels of late-night salivary cortisol, and all had uncontrolled 24-hour urinary cortisol. After 72 weeks of treatment, almost half had normal levels in both the saliva and urine, and nearly 4 in 10 had normal levels in the urine only.
- Improvements in some cardiovascular/metabolic measures were greatest in people who had normal levels of cortisol in both the saliva and urine.
- Physical features generally improved as cortisol levels fell during treatment, even if cortisol levels in the saliva and urine were not completely normalized.
- The greatest improvements in quality of life were seen in people with normal salivary and urinary levels and in those with normal urinary levels.

**What do the results mean?**

- In people with Cushing disease, it may be beneficial to measure cortisol levels in both the saliva and the urine in order to evaluate clinical outcomes, as well as to consider aiming for normalization of both of these measurements for the best patient improvement in a number of evaluations.

**Where can I find more information?**

- LINC 3 primary publication: Pivonello R et al *Lancet Diabetes Endocrinol* 2020;8:748-61.
- LINC 4 primary publication: Gadelha M et al *J Clin Endocrinol Metab* 2022;107:e2882-95.
- LINC 3 long-term data publication: Fleseriu M et al *Eur J Endocrinol* 2022;187:531−41.
- LINC 4 long-term data publication: Gadelha M et al *Front Endocrinol (Lausanne)* 2023;14:1236465.

Cushing disease is a rare disorder caused by endogenous overproduction of cortisol due to an ACTH-producing pituitary adenoma [1, 2]. Physical manifestations of hypercortisolism include weight gain with central obesity, supraclavicular fat pads, proximal myopathy, thinning of the skin, striae, and ecchymoses [3-5]. Comorbid conditions, occurring as a direct result of hypercortisolism, are also common. These include diabetes, dyslipidemia, hypertension and other cardiovascular diseases, osteoporosis, psychiatric disorders, and cognitive impairment [3, 5, 6]. Most patients have reduced health-related quality of life (QoL) arising from the physical symptoms, as well as psychological dysfunction and comorbid burden, and residual QoL impairment may persist despite clinical remission [3, 5-7]. Hypercortisolism is also associated with an increased risk of mortality, primarily as a result of cardiovascular and infectious diseases [3, 8].

The key treatment goals for Cushing syndrome are to normalize cortisol levels, alleviate signs and symptoms, and improve QoL [9, 10]. Cortisol levels in response to treatment are commonly monitored using mean 24-hour urinary free cortisol (mUFC) [9, 11]. Late-night salivary cortisol (LNSC) levels, which reflect the nadir associated with physiological circadian rhythm, may also be useful for assessing treatment response in Cushing disease [9, 12, 13], but to date, the possible association between LNSC control and improvements in signs and symptoms has only been investigated in an exploratory analysis of a pasireotide study and in a small study of ketoconazole and cabergoline combination treatment [11, 14]. In another study, the association between restoration of cortisol circadian rhythm and improved QoL scores was examined in patients with medically treated Cushing disease [13].

Osilodrostat is a potent oral inhibitor of 11β-hydroxylase, the enzyme that catalyzes the final step of cortisol synthesis [15]. As part of the clinical development program, osilodrostat efficacy and safety were demonstrated in 2 large phase III trials in patients with Cushing disease, LINC 3 [16] and LINC 4 [17]. Results from the 48-week core phases showed that osilodrostat provides rapid and sustained reductions in mUFC, alongside improvements in signs, symptoms, and QoL [16, 17]. These biochemical and clinical improvements were maintained during long-term treatment in the extension phases of the studies [18, 19].

The aim of the current analysis was to determine the potential benefits of normalizing both LNSC and mUFC in optimizing outcomes. To achieve this, we pooled outcome data from patients with Cushing disease who received long-term osilodrostat during the LINC 3 and LINC 4 studies. Pooling of the data increased the size of the patient population and, thereby, the number of LNSC and mUFC assessments, as well as allowing evaluation of LNSC and mUFC over an extended treatment period.

## Methods

Details of the LINC 3 and LINC 4 study designs have been published previously [16, 17]. Both were phase III international multicenter studies with a 48-week core phase, followed by an optional extension for patients benefitting from osilodrostat treatment at week 48, as assessed by the study investigator.

### Patients

Patients were eligible for inclusion in the LINC 3 and LINC 4 studies if they were age 18 to 75 years and had either a confirmed diagnosis of persistent/recurrent Cushing disease after pituitary surgery and/or irradiation or de novo disease (nonsurgical candidates). Additional inclusion criteria were morning plasma ACTH above the lower limit of normal (LLN), a confirmed source of excess ACTH from a pituitary origin, and mUFC >1.5 times the upper limit of normal (ULN; LINC 3) or >1.3 × ULN (LINC 4) [16, 17].

The studies were conducted in accordance with the Declaration of Helsinki, with an independent ethics committee/institutional review board at each site approving the study protocols. Patients provided written informed consent to participate at the beginning of the studies and for the extension periods. Each trial is registered at ClinicalTrials.gov (LINC 3, NCT02180217; LINC 4, NCT02697734).

### Study Design

The 48-week core phase of LINC 3 included an 8-week randomized-withdrawal period for eligible patients (weeks 26-34) [16]. All patients received open-label osilodrostat treatment (starting at 2 mg twice daily [bid]), except those randomized to placebo during the 8-week randomized-withdrawal period. Osilodrostat dose titration was permitted every 2 weeks until week 12, then every 4 weeks thereafter.

The 48-week core phase of LINC 4 included an initial 12-week, double-blind, placebo-controlled period, during which patients were randomized to osilodrostat 2 mg bid or matching placebo [17]. Dose titration was permitted every 3 weeks. At week 12, patients restarted osilodrostat 2 mg bid (unless they were on a lower dose at week 12). All patients on <2 mg bid osilodrostat (or matched placebo) at week 12 continued to receive the same dose, regardless of initial treatment allocation. Dose titration was permitted every 3 weeks thereafter.

In both studies, stepwise dose titration (according to the sequence, 2-5-10-20-30 mg bid) was used, with decisions based on efficacy and tolerability. The maximum osilodrostat dose was 30 mg bid in both studies (maximum dose during the first 12 weeks of LINC 4 was 20 mg bid because of the more gradual dose-titration regimen than in LINC 3 [every 3 vs every 2 weeks]). The dose could be reduced if mUFC was below the LLN or in the lower part of the normal range for patients with symptoms of adrenal insufficiency.

### Assessments

#### Cortisol levels

LNSC, mUFC, and morning serum cortisol levels were measured in a central laboratory by liquid chromatography–tandem mass spectrometry (high-throughput liquid chromatography system, Cohesive Technologies; tandem mass spectrometer, ThermoFinnigan). LNSC was determined from a single saliva sample collected between 23:00 and 01:00 in LINC 3 and from 2 saliva samples collected between 22:00 and 23:00 in LINC 4 (normal range, ≤ 2.5 nmol/L [≤0.9 μg/dL]). mUFC was calculated from the mean of 2 or 3 UFC samples (normal range, 11-138 nmol/24 hours [4-50 µg/24 hours]). In LINC 3, patients collected 24-hour urine samples in the 7 days before the next study visit, with the last urine sample preferably collected the day before the visit. In LINC 4, patients collected 2 24-hour urine samples, preferably over the 2 consecutive days immediately before each study visit. Early-morning serum cortisol levels were determined from a single sample (normal range, 127-567 nmol/L [46-206 μg/dL]). mUFC normalization was the primary endpoint in both studies.

#### Cardiovascular/metabolic-related parameters

The following parameters were assessed: systolic blood pressure (SBP) and diastolic blood pressure (DBP), fasting plasma glucose (FPG), weight, body mass index (BMI), waist circumference, total cholesterol, low-density lipoprotein cholesterol, high-density lipoprotein (HDL) cholesterol, and triglycerides.

#### QoL

QoL was assessed using the Cushing Quality of Life Questionnaire (CushingQoL) and the Beck Depression Inventory II (BDI-II). Minimal important differences were defined as an increase of ≥10.1 for CushingQoL scores [20] and a 17.5% reduction in BDI-II scores [21].

#### Physical manifestations of hypercortisolism

Physical manifestations of hypercortisolism, including facial rubor, striae, supraclavicular and dorsal fat pads, proximal muscle atrophy, central obesity, ecchymoses, and hirsutism (females only), were assessed locally from photographs from the shoulders up and of the trunk. These were rated subjectively on a semiquantitative scale: 0 = absent; 1 = mild; 2 = moderate; 3 = severe.

### Safety

Adverse events (AEs) were recorded according to Common Terminology Criteria for Adverse Events and assessed from core study baseline to end of extension. Extensive safety data for the LINC 3 [16, 18] and LINC 4 [17, 19] studies have been reported elsewhere.

### Statistical Methods

Individual patient data from LINC 3 and LINC 4 were pooled and analyzed. To be included in the analyses, patients had to have evaluations of LNSC, mUFC, and the respective clinical/physical feature. Given the differences in study design of the 2 trials, periods during which a patient was randomized to receive placebo (8 weeks in LINC 3 and 12 weeks in LINC 4) were excluded from the pooled analysis.

Changes in cardiovascular/metabolic-related parameters, physical manifestations of hypercortisolism, and QoL were assessed at weeks 48 and 72 in the pooled population according to the following classifications of cortisol control status: both LNSC and mUFC controlled (LNSC ≤ ULN + mUFC ≤ ULN); only mUFC controlled (mUFC ≤ ULN + LNSC > ULN); only LNSC controlled (LNSC ≤ ULN + mUFC > ULN); both LNSC and mUFC uncontrolled (LNSC > ULN + mUFC > ULN). Correlation analyses were performed using Pearson correlation coefficients for data with normal distribution. r coefficients between 0.0 and 0.3 (or 0.0 and −0.3), 0.3 and 0.7 (or −0.3 and −0.7), and 0.7 and 1.0 (or −0.7 and −1.0) represent a weak, moderate, and strong positive (or negative) linear relationship, respectively. Categorical data are presented as frequencies and percentages, and continuous data are presented as mean (SD) or median (min-max) values. Results were analyzed descriptively for all patients with an assessment at both baseline and the given visit. Other than the correlation analyses, no formal statistical testing was performed.

## Results

Overall, 160 patients had baseline LNSC and mUFC data available and were included in the pooled analysis from the core and extension phases of LINC 3 and LINC 4. Of these, 32 discontinued during the core phases (both LNSC and mUFC controlled, n = 13; only LNSC controlled, n = 8; only mUFC controlled, n = 3; both LNSC and mUFC uncontrolled, n = 8) and 41 discontinued during the extension phases (both LNSC and mUFC controlled, n = 15; only LNSC controlled, n = 6; only mUFC controlled, n = 10; both LNSC and mUFC uncontrolled, n = 10).

### Patient Demographics and Baseline Characteristics

The baseline characteristics of the pooled population (Table 1) were typical for patients with Cushing disease and similar to those in the parent studies. Median age was 39.0 years, and 80.6% of patients were female. A high proportion had undergone surgery and/or received previous treatment for their condition. Over 90% had persistent or recurrent disease at LINC study baseline.

**Table 1.** Patient baseline characteristics (LINC 3 and LINC 4 pooled)

	All patients n = 160
Median age, years (min–max)	39.0 (19-70)
Sex, n (%)	
Female	129 (80.6)
Male	31 (19.4)
Race, n (%)	
Caucasian	103 (64.4)
Asian	44 (27.5)
Black	5 (3.1)
Native American	1 (0.6)
Other	4 (2.5)
Unknown	3 (1.9)
Mean weight, kg (SD)	79.7 (19.7)
Mean height, cm (SD)	162.5 (8.6)
Mean BMI, kg/m^2^ (SD)	30.2 (7.2)
Median time to first osilodrostat dose since diagnosis, months (min-max)	54.2 (3-287)
Disease status, n (%)	
De novo	14 (8.8)
Persistent/recurrent	146 (91.3)
Proportion of patients with previous surgery, n (%)	140 (87.5)
Proportion of patients with previous medical treatment for Cushing disease, n (%)	127 (79.4)
Proportion of patients with previous pituitary irradiation, n (%)	22 (13.8)
Proportion of patients with comorbidities, n (%)	149 (93.1)
LNSC, nmol/L	
Mean (SD)	11.7 (18.9); 4.7 (7.5) × ULN
Median (min-max)	7.4 (1-203); 3.0 (0.4-81.2) × ULN
mUFC, nmol/24 hours	
Mean (SD)	759.1 (1300.9); 5.5 (9.4) × ULN
Median (min-max)	374.1 (21-9612); 2.7 (0.2-69.7) × ULN
Proportion of patients ≤ULN at baseline, n (%)	
LNSC only	15 (9.4)
mUFC only*^a^*	5 (3.1)
Both LNSC and mUFC	4 (2.5)

ULN for mUFC is 138 nmol/24 hours (50 μg/24 hours) and for LNSC is 2.5 nmol/L (0.9 ng/mL).

Abbreviations: BMI, body mass index; LNSC, late-night salivary cortisol; mUFC, mean urinary free cortisol; ULN, upper limit of normal.

^*a*^Five patients with mUFC control at baseline had mUFC >138 nmol/24 hours at screening and so met the criteria for study enrollment.

### Osilodrostat Dose and Exposure

In patients with LNSC and mUFC assessments at baseline and at least 1 postbaseline time point, median (min–max) osilodrostat exposure was 97.9 (2-218) weeks (n = 149), median (min–max) average osilodrostat dose was 6.5 (1-47) mg/day, and the mean (SD) dose given for the longest duration was 10.0 (11.9) mg/day.

### Proportion of Patients With LNSC and mUFC Control Over Time and Time to First LNSC and/or mUFC Control

**Figure 1.**
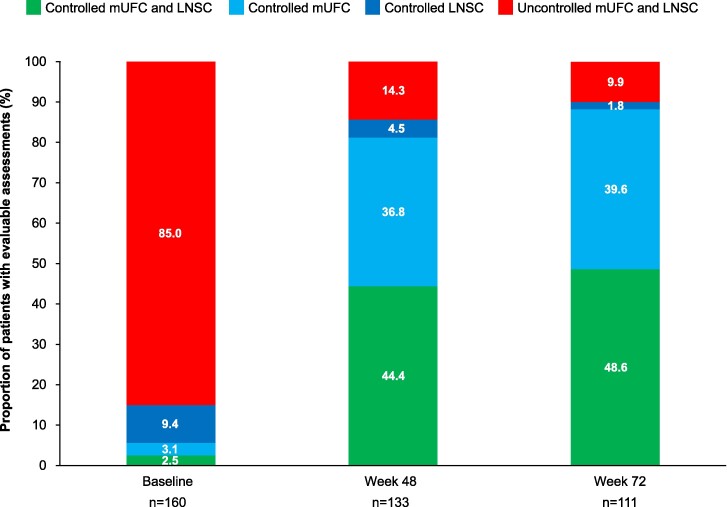
Proportion of patients by LNSC and mUFC control status over time. Abbreviations: LNSC, late-night salivary cortisol; mUFC, mean urinary free cortisol.

Compared with baseline, there was an increase in the proportion of patients with both LNSC and mUFC controlled or only mUFC controlled at week 48, to 44.4% and 36.8%, respectively; these effects were maintained at week 72 (Fig. 1). Median (95% confidence interval) time to first normalization of mUFC, LNSC, and both LNSC and mUFC was 35.0 (34.0-41.0), 82.0 (56.0-84.0), and 335.0 (165.0-504.0) days, respectively. At the time of first LNSC, mUFC, and LNSC and mUFC normalization, osilodrostat dose was ≤10 mg/day in 73.1%, 72.3%, and 68.2% of patients, respectively, >10−≤20 mg/day in 20.8%, 20.3%, and 25.9% of patients, and >20 mg/day in 6.2%, 7.4%, and 5.9% of patients. There was no difference in mUFC or LNSC treatment response observed between patients with de novo and persistent/recurrent disease (data not shown).

### LNSC and mUFC Levels Over Time

**Figure 2.**
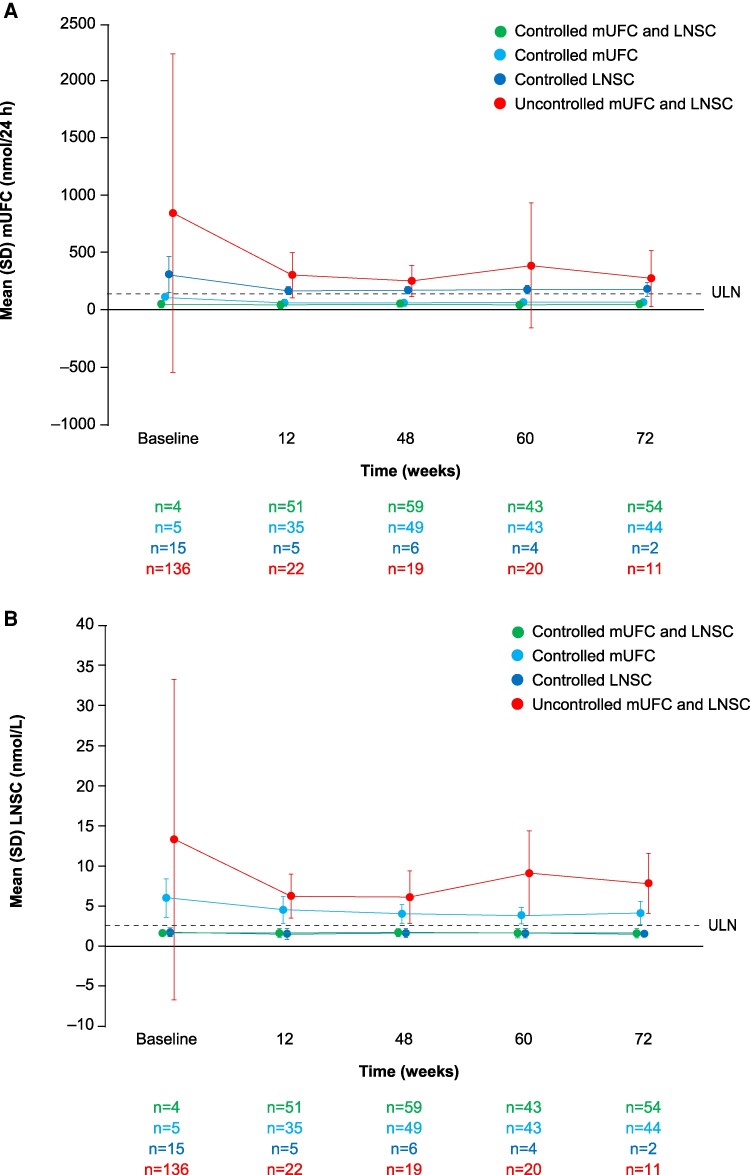
(A) mUFC and (B) LNSC over time by mUFC and LNSC control status. ULN = 2.5 nmol/L (0.9 ng/mL) for LNSC and 138 nmol/24 hours (50 µg/24 hours) for mUFC. Abbreviations: LNSC, late-night salivary cortisol; mUFC, mean urinary free cortisol; ULN, upper limit of normal.

The greatest reductions in mean LNSC and mUFC levels were observed in the group with both LNSC and mUFC levels above normal at baseline (Fig. 2A and 2B). In patients in whom both LNSC and mUFC were normal at baseline, control was maintained during osilodrostat treatment.

**Figure 3.**
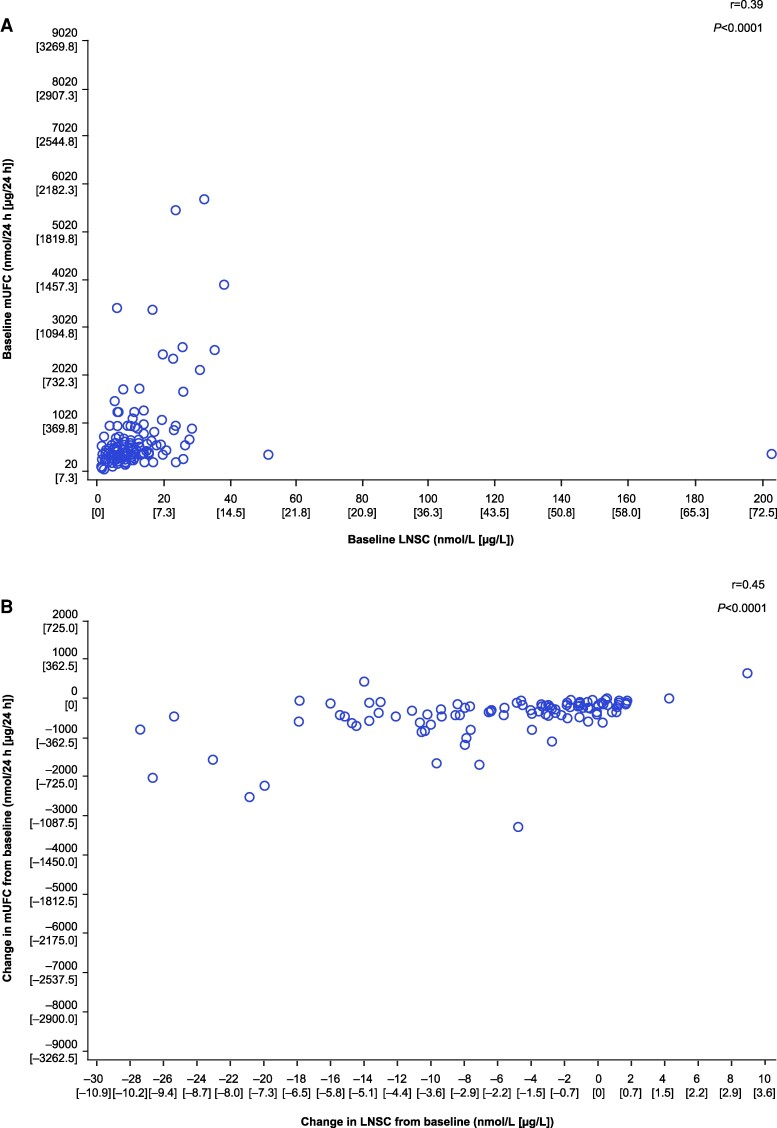
Correlation between (A) baseline LNSC and mUFC levels and (B) changes in LNSC and mUFC levels from baseline to week 72. Abbreviations: LNSC, late-night salivary cortisol; mUFC, mean urinary free cortisol.

There was a moderate positive correlation between LNSC and mUFC levels at baseline (*r* = 0.39, *P* < .0001) and at week 72 (*r* = 0.45, *P* < .0001; Fig. 3). The weak correlation at week 48 was not significant (*r* = 0.18, *P* = .0679).

### Morning Serum Cortisol Levels Over Time

**Figure 4.**
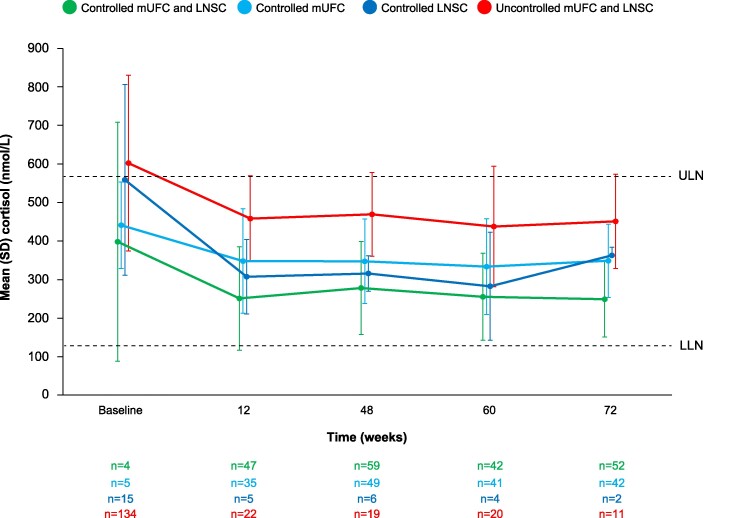
Mean (SD) serum cortisol levels over time by LNSC and mUFC control status. LLN = 127 nmol/L (46.0 ng/mL); ULN = 567 nmol/L (205.5 ng/mL). Abbreviations: LLN, lower limit of normal; LNSC, late-night salivary cortisol; mUFC, mean urinary free cortisol; ULN, upper limit of normal.

Mean morning serum cortisol levels decreased over time in all control status subgroups, although between-patient variability in serum cortisol levels was high (Fig. 4). Mean levels were above the ULN at baseline in the subgroup with both LNSC and mUFC uncontrolled but normalized during osilodrostat treatment. In the other 3 subgroups, mean levels were below the ULN at baseline and were maintained within the normal range during treatment.

### Changes in Cardiovascular and Metabolic-related Parameters

**Figure 5.**
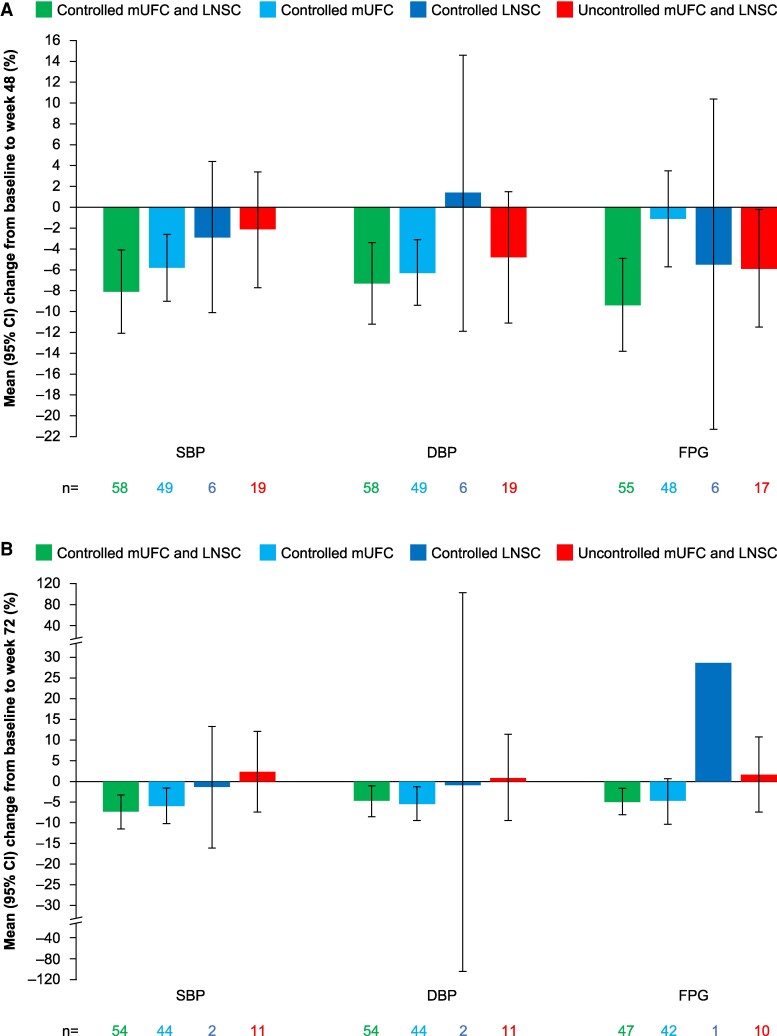
Mean percentage change from baseline to (A) week 48 and (B) week 72 in SBP, DBP, and FPG by LNSC and mUFC control status. Patients with both LNSC and mUFC uncontrolled experienced improvements in cardiovascular and metabolic parameters. Variable n numbers reflect differences in the number of patients with data available for the various parameters. Abbreviations: DBP, diastolic blood pressure; FPG, fasting plasma glucose; LNSC, late-night salivary cortisol; mUFC, mean urinary free cortisol; SBP, systolic blood pressure.

**Figure 6.**
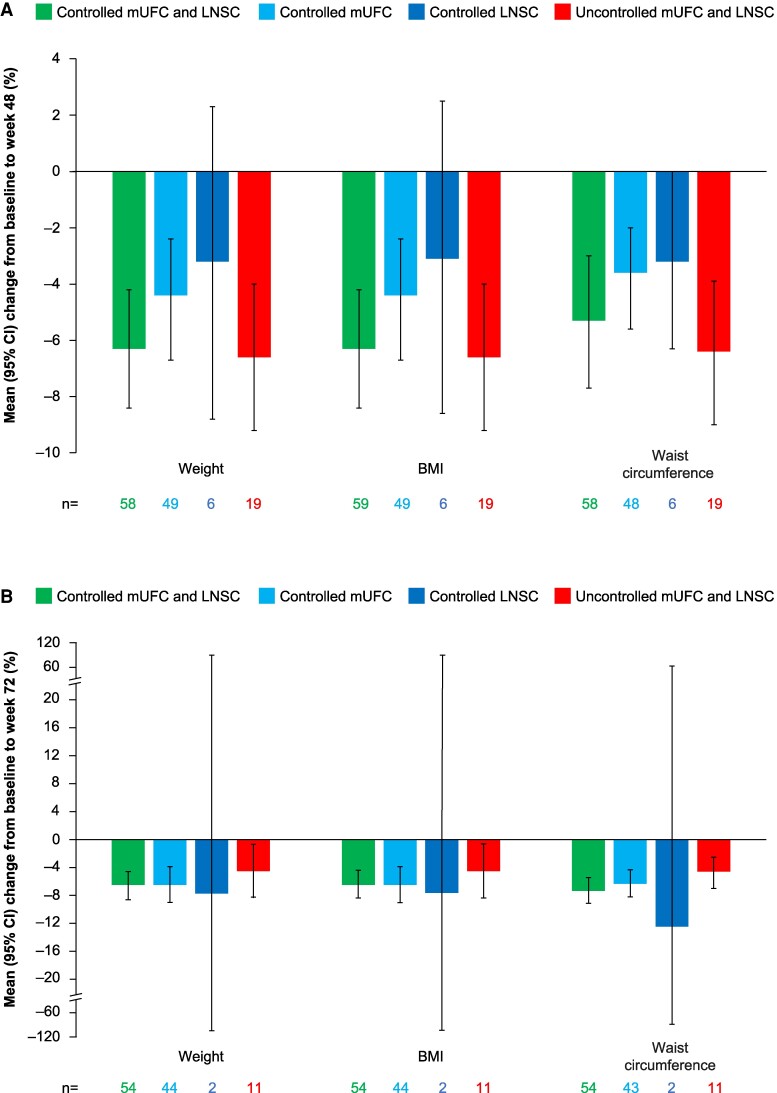
Mean percentage change from baseline to (A) week 48 and (B) week 72 in weight, BMI, and waist circumference by LNSC and mUFC control status. Variable n numbers reflect differences in the number of patients with data available for the various parameters. Abbreviations: BMI, body mass index; CI, confidence interval; LNSC, late-night salivary cortisol; mUFC, mean urinary free cortisol.

There were long-term improvements in SBP, DBP, FPG, weight, BMI, and waist circumference in patients treated with osilodrostat. Patients with both LNSC and mUFC controlled generally had the greatest improvements in blood pressure and glycaemic control (Fig. 5), while improvements in parameters related to body weight were observed in all cortisol control categories (Fig. 6). There was no correlation between time since diagnosis and change from baseline to week 72 in DBP (*r* = −0.02, *P* = .8176) or SBP (*r* = 0.00, *P* = .9673).

**Figure 7.**
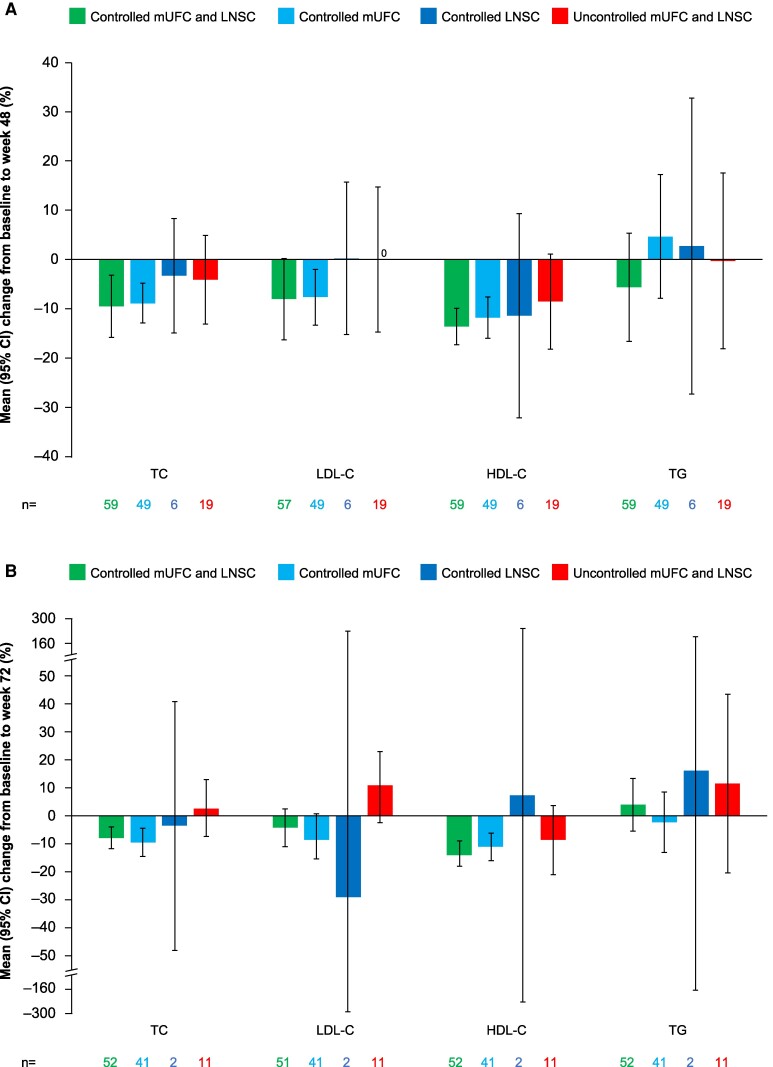
Mean percentage change from baseline to (A) week 48 and (B) week 72 in TC, LDL-C, HDL-C, and TG by LNSC and mUFC control status. Variable n numbers reflect differences in the number of patients with data available for the various parameters. Abbreviations: CI, confidence interval; HDL-C, high-density lipoprotein cholesterol; LDL-C, low-density lipoprotein cholesterol; LNSC, late-night salivary cortisol; mUFC, mean urinary free cortisol; TC, total cholesterol; TG, triglycerides.

There were also long-term improvements in total cholesterol and low-density lipoprotein cholesterol in patients with both LNSC and mUFC controlled and in patients with only mUFC controlled (Fig. 7). HDL cholesterol decreased at weeks 48 and 72; this was generally irrespective of cortisol control category. Triglycerides remained largely unchanged during the parent studies.

There were no moderate or strong correlations between change from baseline to week 72 in mUFC or LNSC levels and changes in cardiovascular and metabolic-related parameters, with the exception of FPG and HDL cholesterol, which appear to have a weak to moderate correlation with change in mUFC and LNSC (Table 2).

**Table 2.** Correlation coefficients for change in LNSC/mUFC and change in cardiovascular and metabolic-related parameters at week 72

Change in parameter	Change in LNSC	Change in mUFC
SBP	*r* = 0.08 *P* = .4686	*r* = 0.18 *P* = .0341
DBP	*r* = −0.03 *P* = .7757	*r* = 0.18 *P* = .0283
FPG	*r* = 0.39 *P* = .0003	*r* = 0.27 *P* = .0089
HbA_1c_	*r* = 0.21 *P* = .0428	*r* = 0.21 *P* = .0325
Weight	*r* = 0.05 *P* = .6284	*r*=−0.00 *P* = .9659
BMI	*r* = 0.03 *P* = .7465	*r*=−0.00 *P* = .9593
Waist circumference	*r* = 0.28 *P* = .0063	*r* = 0.09 *P* = .3673
TC	*r* = 0.04 *P* = .6925	*r* = 0.12 *P* = .2416
LDL-C	*r* = 0.05 *P* = .6540	*r* = 0.00 *P* = .9805
HDL-C	*r* = 0.14 *P* = .1889	*r* = 0.31 *P* = .0013
TG	*r* = −0.15 *P* = .1687	*r* = 0.05 *P* = .6115

Abbreviations: BMI, body mass index; DBP, diastolic blood pressure; FPG, fasting plasma glucose; HbA_1c_, glycated hemoglobin; HDL-C, high-density lipoprotein cholesterol; LDL-C, low-density lipoprotein cholesterol; LNSC, late-night salivary cortisol; mUFC, mean urinary free cortisol; SBP, systolic blood pressure; TC, total cholesterol; TG, triglycerides.

### Changes in Physical Manifestations of Hypercortisolism

**Figure 8.**
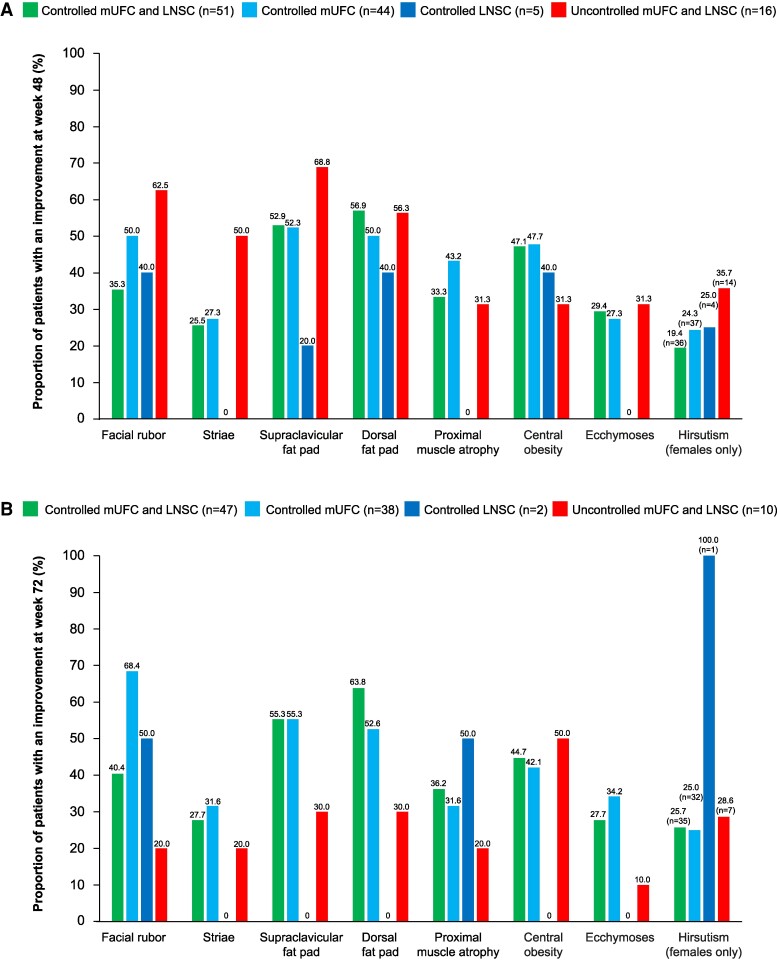
Proportion of patients with improvements from baseline to (A) week 48 and (B) week 72 in physical manifestations of hypercortisolism by LNSC and mUFC control status. Abbreviations: LNSC, late-night salivary cortisol; mUFC, mean urinary free cortisol.

There were improvements in physical manifestations of hypercortisolism irrespective of LNSC or mUFC control, and these improvements were maintained over long-term treatment (Fig. 8).

### Changes in CushingQoL and BDI-II Scores

**Figure 9.**
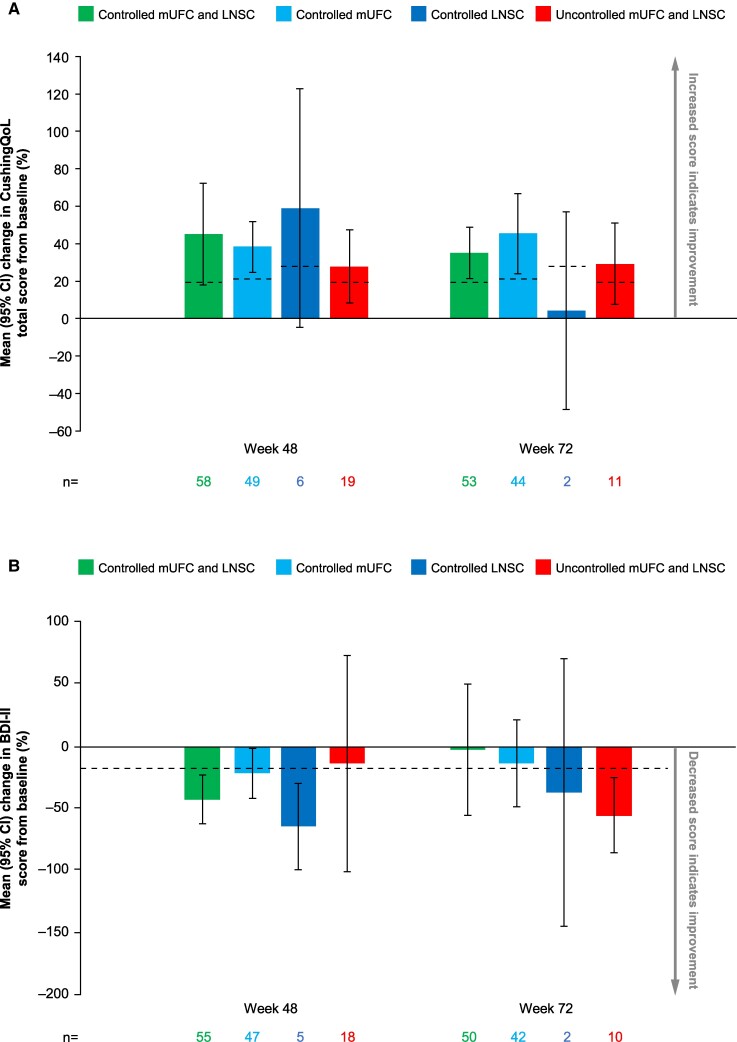
Mean percentage change from baseline to weeks 48 and 72 in (A) CushingQoL and (B) BDI-II scores by LNSC and mUFC control status. Dashed lines indicate the minimal important difference of (A) 10.1 points in CushingQoL score (corresponding to a percentage change of 19.9% for both LNSC and mUFC controlled, 21.1% for only mUFC controlled, 27.7% for only LNSC controlled, and 19.5% for both LNSC and mUFC uncontrolled) and (B) −17.5% in BDI-II score. Abbreviations: BDI-II, Beck Depression Inventory II; CI, confidence interval; CushingQoL, Cushing Quality of Life Questionnaire; LNSC, late-night salivary cortisol; mUFC, mean urinary free cortisol.

Changes in CushingQoL and BDI-II scores from baseline to week 48 and from baseline to week 72 are shown in Fig. 9. Patients with both LNSC and mUFC controlled or only mUFC controlled generally had the greatest improvements in CushingQoL scores, which were maintained during long-term treatment. At week 72, mean percentage changes were above the minimal important difference of 10.1 points (corresponding to a percentage change of 19.9% for both LNSC and mUFC controlled, 21.1% for only mUFC controlled, 27.7% for only LNSC controlled, and 19.5% for both LNSC and mUFC uncontrolled) in all cortisol control subgroups except only LNSC controlled (n = 2).

Improvements in BDI-II scores were observed and were generally maintained for most patients during long-term treatment, regardless of LNSC or mUFC control. At week 72, mean percentage changes were above the minimal important difference of −17.5% in the subgroup with only LNSC controlled and the subgroup with both LNSC and mUFC uncontrolled.

At week 72, there were weak correlations between change in CushingQoL score and change in LNSC and mUFC (*r* = −0.27, *P* = .0084 and *r* = −0.26, *P* = .0061, respectively) and between change in BDI-II score and change in LNSC and mUFC (*r* = 0.33, *P* = .0011 and *r* = 0.24, *P* = .0110, respectively).

#### AEs

In general, the proportion of patients with reported AEs in the pooled safety set and the number of AEs reported decreased over time (Table 3). The most persistent AEs reported (Table 3) were fatigue (weeks 0-12, 19.5%; weeks 12-48, 18.5%; weeks 48-72, 11.2%; week 72 onwards, 12.6%) and nausea (weeks 0-12, 24.8%; weeks 12-48, 17.1%; weeks 48-72, 0.0%; week 72 onwards, 14.3%).

**Table 3.** Proportion of patients with reported AEs in the pooled safety set and most commonly reported (≥10% of patients) AEs over time

Patients, n (%)	Weeks 0-12 n = 149	Weeks 12-48 n = 146	Weeks 48-72 n = 134	Week 72 onwards n = 119
Any AEs	116 (77.9)	139 (95.2)	98 (73.1)	96 (80.7)
Nausea	47 (24.8)	25 (17.1)	−	17 (14.3)
Fatigue	29 (19.5)	27 (18.5)	15 (11.2)	15 (12.6)
Decreased appetite	27 (18.1)	17 (11.6)	—	—
Arthralgia	25 (16.8)	27 (18.5)	—	—
Adrenal insufficiency	23 (15.4)	17 (11.6)	—	—
Diarrhea	17 (11.4)	—	—	—
Headache	17 (11.4)	30 (20.5)	—	—
Myalgia	17 (11.4)	18 (12.3)	—	—
Asthenia	16 (10.7)	—	—	—
Peripheral edema	15 (10.1)	—	—	—
Back pain	—	17 (11.6)	—	—
Hypertension	—	16 (11.0)	—	—
Increased blood testosterone	—	16 (11.0)	—	—
Dizziness	—	15 (10.3)	—	—

Abbreviation: AE, adverse event.

## Discussion

Hypercortisolism in patients with Cushing syndrome is associated with a range of comorbid conditions and an increased risk of mortality [3, 22]. The key goal of treatment is to normalize cortisol levels, thus reducing comorbidities and the risk of mortality [8, 9]. This analysis highlights the importance of combined LNSC and mUFC control in order to achieve optimal long-term clinical benefits in patients with Cushing disease. For patients in whom both of these parameters were normalized, percentage improvements from baseline in cardiovascular/metabolic-related parameters (SBP, DBP, and FPG) were generally greater than in patients with only mUFC controlled or both LNSC and mUFC uncontrolled. Long-term improvements in cardiovascular/metabolic-related parameters have important implications for all patients with Cushing disease, underscoring the importance of controlling both LNSC and mUFC. These include the potential to alleviate the burden of comorbidities, discontinue (or at least reduce the dose of) concomitant medication, and reduce the risk of mortality.

The observation that there was no correlation between time since diagnosis and change in blood pressure suggests that patients benefit from treatment regardless of the duration of hypercortisolism. Interestingly, improvements from baseline in weight, BMI, and waist circumference were observed in all groups that achieved control of 1 or both cortisol parameters, and improvements in many physical manifestations of hypercortisolism were observed during long-term treatment regardless of whether cortisol was controlled; this may be reflective of a decrease in cortisol levels from high baseline levels. In the subgroup with both LNSC and mUFC uncontrolled, this likely reflects the reduction in severity of hypercortisolism in these patients, with LNSC, mUFC, and serum cortisol all being reduced to much lower levels. Patients with both LNSC and mUFC controlled, or only mUFC controlled, also had the greatest improvement from baseline in CushingQoL scores. It is notable that improvements in BDI-II scores were greater in those with LNSC control alone than in those with mUFC control alone, given that disturbances in the circadian rhythm of cortisol secretion (as indicated by high LNSC levels) are associated with depression, and restoring normal circadian rhythm may improve the symptoms of depression [23]. However, a study in patients with medically treated Cushing disease found that there was no significant difference in QoL scores between those who achieved recovery of cortisol diurnal rhythm and those who did not, although treatment duration was short (80 days) [13]. Interpretation of the results from the current study is limited by the relatively small number of patients with LNSC control only, and further evaluation of LNSC normalization and the effect on symptoms of depression is needed in a larger patient group.

At weeks 48 and 72, most patients (>80%) had either controlled LNSC and mUFC or controlled mUFC only. Interestingly, there was no difference in treatment response observed between patients with de novo and persistent/recurrent disease. Time to first normalization was 35.0 days for mUFC, 82.0 days for LNSC, and 335.0 days for both LNSC and mUFC. This time course reflects the increased stringency of criteria evaluating control; it is unsurprising that this was accompanied by a longer period before achieving control of both LNSC and mUFC. mUFC evaluates cortisol by integrating the effect of multiple cortisol pulses throughout a 24-hour period, whereas LNSC takes a measurement at a single time point during the late-night nadir [24]. mUFC measurements may therefore be more useful when evaluating overall treatment response to steroidogenesis inhibition, while LNSC, taken at a single time point, is more sensitive to disruption of circadian rhythm and may be a good marker of long-term treatment response. A potential physiological basis for the observed longer time to normalization of LNSC than of mUFC could be the length of time needed for the hypothalamus–pituitary–adrenal axis to reestablish its feedback mechanisms and normalize the circadian rhythm of cortisol production [25] following reduced cortisol levels from steroidogenesis inhibition. At the time of normalization, most patients were on doses of osilodrostat of ≤10 mg bid, consistent with the usual maintenance doses seen across clinical trials (2-7 mg bid) [9]. In the context of the 12% to 19% of patients not achieving mUFC normalization, findings from the LINC 3 study are interesting as they show that improvements in clinical signs and symptoms and physical manifestations of hypercortisolism can be achieved even in patients with partial control of mUFC [26]. LNSC normalization and monitoring are paramount in selected patients with Cushing disease; for example, LNSC is more likely to show abnormal findings before UFC does in patients with recurrent disease, potentially allowing earlier intervention [9, 27]. In the current analysis, the proportion of patients with only LNSC controlled was small (<10%) and did not improve during osilodrostat treatment. This may reflect the fact that in many patients, only 1 LNSC sample was evaluated at each time point in LINC 3; more regular assessment of multiple LNSC samples over time may provide more accurate results. It is also possible that, for some patients, using a higher osilodrostat dose at night (compared with the morning dose) could result in higher rates of LNSC normalization, although clinical data are required to confirm this [9, 28]. It is notable that, in the current analysis, there were trends for long-term reductions in LNSC in patients with mUFC control. As Cushing disease is chronic in nature, lifelong monitoring is required; therefore, long-term safety and effectiveness data from osilodrostat treatment in clinical practice would be helpful in discerning the sustainability of the clinical improvements outlined previously and identifying any potential late-emerging AEs. The LINC 3 study extension had a median (range) exposure time of 130 (1-245) weeks and demonstrated the long-term efficacy and tolerability of osilodrostat in this patient population, with no new safety signals reported [18]. To build on this, studies with longer treatment duration and larger patient numbers would be useful to provide insight into safety and efficacy during chronic osilodrostat administration. The incidence of AEs generally decreased over time, and they were less frequent from week 48 onwards; however, reports of nausea and fatigue persisted during long-term treatment.

AEs related to the pharmacology of osilodrostat have been previously reported in patients with Cushing disease and should be monitored throughout treatment [16-19]. This should include educating patients on the symptoms of hypocortisolism, including of glucocorticoid withdrawal (mood disturbances, hypersomnia, decreased appetite, weight loss, loss of muscle mass, myalgia, and fatigue [29]) and adrenal insufficiency (hypotension, hypoglycemia, decreased appetite, vomiting, weight loss, myalgia, and fatigue [29]). It should be noted that in the LINC clinical trials, hypocortisolism-related AEs occurred mostly during dose titration but can occur at any time during treatment [16-19]. Patients should also be monitored for signs and symptoms indicating the accumulation of adrenal hormone precursors or subsequent increases in androgen levels (elevated blood testosterone in women, acne, hirsutism in women, peripheral edema, hypertension, and hypokalemia [17]) and for QT-interval prolongation or changes in arrhythmogenic potential.

Cortisol and cortisone (the inactive form generated from cortisol) can also be measured in hair samples, in addition to saliva and urine. In the Haircush study, patients on medical treatment for Cushing disease with normalized UFC had significantly higher hair cortisone levels than patients who had achieved remission for at least 1 year after surgery; they also had significantly higher clinical scores (adapted from an arbitrary scale used in a previous cabergoline study [30]), UFC, and late-night salivary cortisone. In addition, patients on medical treatment with increased hair cortisone levels required significantly higher doses of antihypertensive medication than those with normal hair cortisone levels [31]. These data support the assertion that normalization of UFC alone is an insufficient biomarker for disease control.

Various other medical treatments are available for the management of Cushing disease. These include other steroidogenesis inhibitors (eg, ketoconazole, levoketoconazole, metyrapone), somatostatin receptor ligands (pasireotide), dopamine receptor agonists (cabergoline), and glucocorticoid receptor blockers (mifepristone, when hyperglycemia is also present) [9]. Consistent with the results of a previous pasireotide study in patients with Cushing disease, there was a moderate correlation between LNSC and mUFC during the 12-month treatment period [12]. In another study, however, there was no clear direct association between normalization of mUFC and reestablishment of cortisol diurnal rhythm (defined as midnight serum and salivary cortisol levels <75% of the 09:00 value) in medically treated patients with Cushing disease [13].

From baseline to week 72, mUFC levels were generally lower in patients with both LNSC and mUFC normalized than in those with mUFC normalization alone; in this second group, mUFC values were closer to the ULN, perhaps indicating that patients with lower mUFC levels (toward the middle of the normal range) could potentially achieve LNSC normalization. The link between control of both LNSC and mUFC and treatment outcomes has only been explored in 2 previous studies, 1 evaluating pasireotide [11] and the other evaluating the combination of cabergoline with ketoconazole [14]. The former was based on an exploratory analysis of data from a phase III study and showed that improvements in SDP, DBP, and body weight were greater in those with dual control than in those with control of only 1 parameter [11]. The authors concluded that simultaneous control of both LNSC and UFC is likely to be an important treatment goal for patients with Cushing disease and that care should be taken when assessing the efficacy of any given treatment option based on control of LNSC or UFC alone. Similar results were reported in the study on cabergoline plus ketoconazole, with greater improvements in blood pressure seen in patients who had both LNSC and UFC levels normalized during treatment than in those who had only UFC normalized [14]. In the phase III SONICS and LOGICS studies of levoketoconazole, mUFC normalized in 31% [32] and 50% [33] of patients after 6 months of maintenance therapy and 2 months of randomized withdrawal, respectively. In both studies, some numerical improvements in the signs and symptoms of Cushing syndrome were also observed, but neither study investigated improvements in signs and symptoms according to LNSC and mUFC control status [32, 33]. In the 6-month SONICS extension, 61% had normalized mUFC at extension entry, which was maintained in 41% of patients after 12 months of treatment; however, no consistent influence on cortisol diurnal rhythm was demonstrated as mean LNSC change was only significant (*P* = .028) at month 6 [34]. Aiming for both LNSC and mUFC control during medical therapy is recommended wherever possible [35]; these data, alongside the results of this analysis, suggest that this may confer the greatest treatment benefit in patients with Cushing disease.

Limitations of the current analysis include differences in levels of osilodrostat exposure between LINC 3 and LINC 4 at various time points because of differences in study design (specifically, the timing of the placebo-controlled periods and the titration schedules used). Also, the primary endpoint of both studies was mUFC normalization, and osilodrostat dose adjustments were based only on this and not on LNSC normalization. Another limitation is that, for many patients, only a single LNSC sample was taken at each time point, while for mUFC, several samples were available at each point; the most recent consensus guidelines recommend that at least 2 LNSC samples be taken [9]. A further limitation is the small number of patients in some groups, despite the pooled analysis increasing the size of the patient population and allowing evaluation of an extended treatment period. In particular, very few patients had control of LNSC only. Interindividual variability in both UFC and LNSC is high [9]; therefore, larger group sizes are needed to provide more robust results. It would also be of interest for future studies to evaluate the relationship between controlling both mUFC and LNSC and the incidence of hypocortisolism-related events.

## Conclusions

Based on a pooled analysis of data from 2 phase III studies of osilodrostat in patients with Cushing disease, normalization of both LNSC and mUFC generally had the greatest improvements in cardiovascular/metabolic-related parameters and QoL compared with patients with only mUFC or LNSC controlled, or both LNSC and mUFC uncontrolled, suggesting that normalization of mUFC alone is not a sufficient marker of optimal disease control. In contrast, improvements in weight, BMI, and waist circumference were observed in all groups that achieved control of 1 or both cortisol parameters, while improvements in most physical manifestations of hypercortisolism were observed regardless of cortisol control, likely because of overall lower cortisol levels. These data show that control of both LNSC and mUFC is associated with improvement in some long-term treatment outcomes beyond that observed with mUFC normalization alone in patients with Cushing disease. Individualized treatment strategies should therefore include the goal of normalizing both LNSC and mUFC while avoiding adrenal insufficiency.

## Data Availability

The datasets generated and analyzed for the current study are not publicly available but are available from the corresponding author on reasonable request. Recordati Rare Diseases will share the complete deidentified patient dataset, study protocol, statistical analysis plan, and informed consent form upon request, effective immediately following publication, with no end date.
